# Bright Quantum Dot Single-Photon Emitters at Telecom Bands Heterogeneously Integrated on Si

**DOI:** 10.1021/acsphotonics.2c00027

**Published:** 2022-06-22

**Authors:** Paweł Holewa, Aurimas Sakanas, Ugur M. Gür, Paweł Mrowiński, Alexander Huck, Bi-Ying Wang, Anna Musiał, Kresten Yvind, Niels Gregersen, Marcin Syperek, Elizaveta Semenova

**Affiliations:** † Laboratory for Optical Spectroscopy of Nanostructures, Faculty of Fundamental Problems of Technology, Department of Experimental Physics, Wrocław University of Science and Technology, Wyb. Wyspiańskiego 27, 50-370 Wrocław, Poland; ‡ DTU Fotonik, Technical University of Denmark, Kongens Lyngby 2800, Denmark; ¶ DTU Electrical Engineering, Technical University of Denmark, Kongens Lyngby 2800, Denmark; § Center for Macroscopic Quantum States (bigQ), Department of Physics, Technical University of Denmark, Kongens Lyngby 2800, Denmark; ∥ Hefei National Laboratory for Physical Sciences at Microscale, University of Science and Technology of China, Hefei, Anhui 230026, China; ⊥ NanoPhoton-Center for Nanophotonics, Technical University of Denmark, Kongens Lyngby 2800, Denmark

**Keywords:** semiconductor quantum dots, InAs/InP, heterogeneous integration, telecom spectral range, single-photon sources, photon extraction efficiency

## Abstract

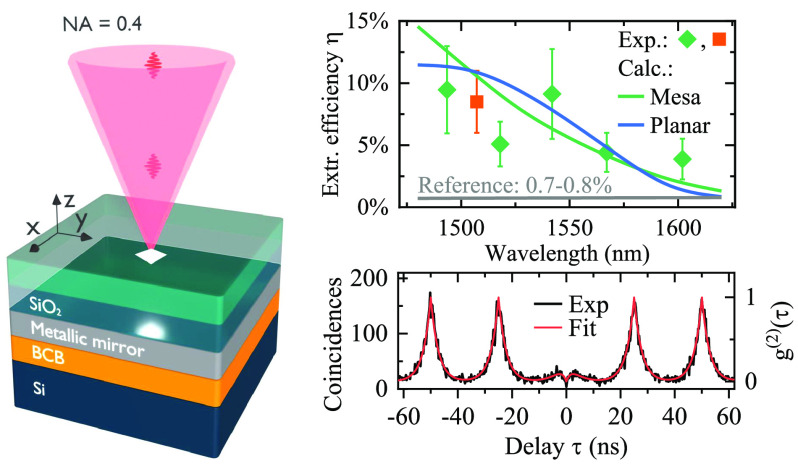

Whereas the Si photonic platform is highly attractive for scalable optical quantum information processing, it lacks practical solutions for efficient photon generation. Self-assembled semiconductor quantum dots (QDs) efficiently emit photons in the telecom bands (1460–1625 nm) and allow for heterogeneous integration with Si. In this work, we report on a novel, robust, and industry-compatible approach for achieving single-photon emission from InAs/InP QDs heterogeneously integrated with a Si substrate. As a proof of concept, we demonstrate a simple vertical emitting device, employing a metallic mirror beneath the QD emitter, and experimentally obtained photon extraction efficiencies of ∼10%. Nevertheless, the figures of merit of our structures are comparable with values previously only achieved for QDs emitting at shorter wavelength or by applying technically demanding fabrication processes. Our architecture and the simple fabrication procedure allows for the demonstration of high-purity single-photon generation with a second-order correlation function at zero time delay, *g*^(2)^(τ = 0) < 0.02, without any corrections at continuous wave excitation at the liquid helium temperature and preserved up to 50 K. For pulsed excitation, we achieve the as-measured *g*^(2)^(0) down to 0.205 ± 0.020 (0.114 ± 0.020 with background coincidences subtracted).

## Introduction

Exploiting single photons as a resource is a powerful approach for quantum information processing (QIP).^1−4^ Photons have long coherences and efficiently propagate over macroscopic distances, which enabled the demonstration of a computational advantage with a quantum photonic processor,^5^ loophole-free tests of Bell’s theorem,^6,7^ and long-distance quantum key distribution.^8,9^ Scalability of optical QIP requires the miniaturization, coupling, and integration of active and passive photonic components into quantum photonic integrated circuits (QPICs).^10^ The Si-based photonic platform is a leading candidate for integration with transparency wavelengths greater than 1.1 μm and mature manufacturing processes.^4,11^ QPICs supporting multidimensional entanglement^11^ and quantum processors with hundreds of elements^11,12^ have been demonstrated. Si however does not allow for efficient light generation. Spontaneous four-wave mixing is the commonly employed approach for single-photon generation on Si and realized with Si-on-insulator integration.^11−13^ This process is probabilistic with few-percent efficiency,^10^ thus limiting scalability. Hybrid approaches combining solid-state single-photon emitters (SPEs) with Si have instead been investigated,^14^ but so far requiring technically demanding fabrication. The reliable realization of SPEs monolithically integrated with Si and allowing for the deterministic emission of pure single photons remains challenging.

Among different candidates,^15,16^ epitaxially grown self-assembled semiconductor quantum dots (QDs)^17^ emitting in the long-wavelength telecom bands (1460–1625 nm)^18^ are suitable for integration with Si.^11,19,20^ The telecom wavelength promises very low Si-waveguide propagation losses^19^ and allows for interconnecting distant QIPCs using optical fiber networks and for distributed quantum computing.^21,22^ Photon emission in the telecom bands has been achieved with QDs based on either InAs/GaAs^23−27^ or InAs/InP^28−36^ material composition, and excellent quantum light sources with high purity^29^ were demonstrated, fulfilling the requirements for QIP.^17,18,37^

The photon extraction efficiency η for as-grown QD-based SPEs is typically <1%^17^ due to the large semiconductor–air refractive index contrast, but can be increased by tailoring the local optical environment.^38−40^ Common approaches for increasing η include placing a QD in a monolithic cavity defined by distributed Bragg reflectors (DBRs),^33^ in an optical horn structure,^28^ or atop a single DBR reflector.^24,25,31,41^ DBR-based approaches are scalable and η up to 13% was achieved in a narrow spectral window,^41^ following demanding fabrication in the InAs/InP material system due to the layers’ low refractive index contrast. With the horn structure, η ≈ 11% at 1560 nm (numerical aperture NA = 0.55) was shown,^28^ requiring complex fabrication. These approaches however are not suitable for the monolithic integration of QD-based SPEs with Si.

In this work, we propose and demonstrate efficient single-photon emission with η > 10% (NA = 0.4) and wavelength in the telecom bands. Our photon sources are based on InAs QDs epitaxially grown on InP and heterogeneously integrated on a Si substrate via chemical bonding. We achieve triggered single-photon emission with *g*^(2)^(0) < 0.02 up to 50 K. A further increase of η is possible employing a higher NA objective and tailoring the QD optical environment. Our approach promises localizing individual QDs via optical imaging^42^ and subsequent processing of photonic components with deterministic spatial alignment. Moreover, our QD integration method on the Si platform provides a broad range of device architecture possibilities, in particular the in-plane emission into planar waveguides as required for on-chip integration.

## Results

### Structure Design

**Figure 1:**
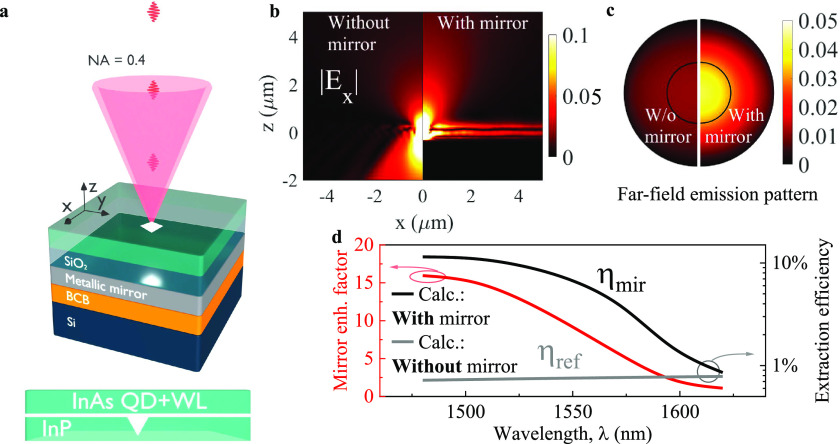
Design of our structures and theoretically estimated performance. (a) Investigated structure scheme, consisting of InAs/InP quantum dots (QDs) with a metallic reflector integrated on a Si substrate. WL, wetting layer. (b) The electric field component |*E*_*x*_| for λ = 1550 nm for the structure without (left) and with (right) a metallic reflector made of aluminum (Al). (c) Calculated far field emission (*P*_lens,NA_) for the reference (left) and the device with an Al mirror (right). The half-circle marks the collection cone of a 0.4 NA objective. (d) Calculated broadband mirror enhancement factor (left axis) and photon extraction efficiency for the QD device with a mirror (η_mir_) and the reference structure without a mirror (η_ref_) as a function of emitter wavelength.

The schematic design of our structures together with calculated device performances are presented in Figure 1. Self-assembled InAs QDs are placed in a weak planar cavity system formed between a bottom metallic mirror and a top InP/air interface, as illustrated in Figure 1a. We determine the achievable photon extraction efficiency using numerical simulations of the electromagnetic (EM) field (Figure 1a) generated by the InAs QD modeled as a classical point dipole with in-plane dipole orientation. The EM field distribution (|*E*_*x*_|) is presented in Figure 1b for a reference structure (as-grown InAs/InP QDs) without (left panel) and with a metal mirror (right panel). We observe that the effect of the mirror is to suppress leakage of light into the substrate and instead direct light in the vertical direction. While additional in-plane guiding of light in the slab waveguide formed by the air–semiconductor–metal interfaces is visible, the out-of-plane field pattern is significantly enhanced compared to the structure without a reflector. Furthermore, the enhancement is observed in the far field emission pattern presented in Figure 1c, highlighting the role of the metallic mirror for the directional emission. The black circle represents the collection aperture of a typical, long-working-distance microscope objective with a 0.4 NA used for light collection in the experiment, and the extraction efficiency η is then defined as the ratio of the power collected within the NA of the objective (*P*_lens,NA_) to the total power emitted by the dipole. The computed extraction efficiency is presented in Figure 1d as a function of wavelength. We define the mirror enhancement factor as the ratio between the extraction efficiency for the planar structure with a mirror (η_mir_) and the reference structure (η_ref_). The presence of the metallic reflector leads to a broadband enhancement of the extraction efficiency (left axis), with the 9.2-fold increase at 1550 nm and η ≈ 7% and nearly 16-fold increase at 1500 nm and η ≈ 11%. This expected performance is competitive with state-of-the-art extraction efficiencies^28,41^ for single-photon sources operating in the long-wavelength telecom bands.

The device fabrication begins with the epitaxy of an InGaAs sacrificial layer lattice-matched to a standard (001)-oriented InP substrate, followed by the growth of an InP λ–cavity with an array of low surface density (∼2.8 × 10^9^ cm^–2^) InAs QDs placed in the center for quantum confinement. In the next step, the top InP surface is covered by 100  nm of SiO_2_ followed by a 100-nm-thick metallic reflector (Al in our case). Subsequently, the chip is flipped and bonded to a Si substrate using benzocyclobutene (BCB), and finally, the thick InP substrate, now on top, and the InGaAs sacrificial layer are removed. We emphasize that this approach and the dimensions are suitable for in-plane photon emission into a Si photonic circuit, although not explicitly pursued in this work.

**Figure 2:**
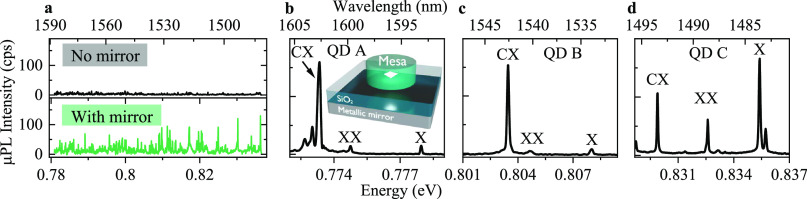
Excitonic complexes in InAs/InP QDs. (a) Representative high spatially resolved photoluminescence (μPL) spectra recorded for the reference structure without a mirror (top panel) and the structure with a mirror (bottom panel) with identical pulsed laser excitation at *T* = 4.2 K. (b–d) μPL spectra of the investigated InAs/InP QDs (labeled A, B, and C) with identified excitonic emission complexes: neutral exciton (X), biexciton (XX), and charged exciton (CX). Inset in (b): mesa structure.

We applied a 2-fold experimental evaluation strategy to verify the significant robustness of the structure design with respect to the level of η and the related broadband performance and to present bright SPEs heterogeneously integrated on a Si substrate. In Figure 2a, we present typical emission spectra obtained from a reference structure without a metallic reflector and the planar structure containing the metallic mirror, both recorded with the same high spatial resolution photoluminescence setup (μPL) from a diffraction-limited spot. The spectra consist of multiple sharp emission lines distributed over a broad spectral range, originating from QDs of mainly different sizes within the optical excitation spot and various emission complexes including neutral exciton (X), charged exciton (CX), and biexciton (XX) transitions. For the structure containing the mirror and compared to the reference structure, we observe a clear intensity enhancement of the emission lines. The quantitative analysis of photon extraction efficiency η from SPEs requires the isolation of single QDs and identification of their respective spectral features. We therefore proceeded with the processing of the mirror-containing planar structure to fabricate cylindrical mesas with diameters of *D*_1_ = 2 μm and *D*_2_ = 3 μm, respectively, as schematically illustrated in the inset of Figure 2b. The finite size of the mesas allows for the spatial isolation of single QDs, a vital element in single-photon source engineering, and leads to modifications of the calculated EM field pattern and extraction efficiencies. In Figures 2b–2d, we present the μPL spectra of three representative and isolated QD emitters, which in the following we refer to as QD A, B, and C, with their emission spectrum located in the telecom L-, C-, and S-band, respectively. The indicated excitonic complexes (X, CX, XX) are identified based on a series of excitation power-dependent and polarization-resolved μPL measurements and confirmed by the cross-correlation of the XX–X and CX–X complexes.

**Figure 3:**
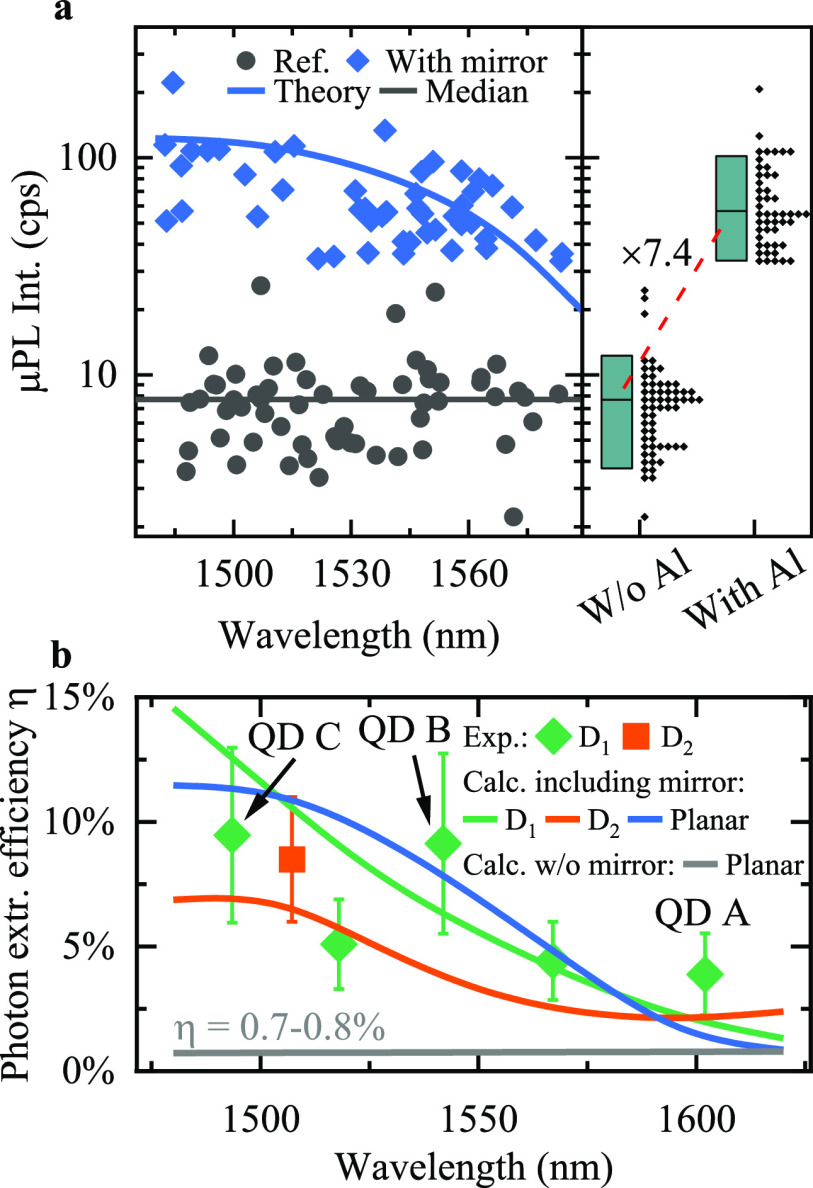
Photon extraction efficiency for the investigated structures. (a) Left panel: comparison of the μPL intensity of ∼50 of the brightest emission lines (points) for the planar mirror-containing (blue diamonds) and the reference (black circles) structures, respectively. The solid blue line is the expected μPL intensity for the mirror-containing structure obtained by multiplying the median μPL intensity of the reference structure (solid black line) by the mirror enhancement factor (cf. red line in Figure 1d). Right panel: statistical analysis of measured intensities. Boxes illustrate one standard deviation; the line inside the box is the median value of each distribution shown as points. (b) Photon extraction efficiency η for the mesa-processed structure with a metallic mirror. Green diamonds show recorded η values for mesas with *D*_1_ = 2 μm including QDs A–C. The result shown as an orange square is obtained for an emitter in a mesa with *D*_2_ = 3 μm. The solid lines represent calculated η values obtained with the modal method for mesas with *D*_1_ = 2 μm (green) and *D*_2_ = 3 μm (orange). Solid blue and gray lines show the calculated η for a planar structure with a mirror and a reference structure without a mirror, respectively.

### Brightness of SPEs

We employ two approaches to compare the calculated with the experimentally obtained photon extraction efficiency η. In the first approach, we consider the broadband enhancement of the photon extraction for planar structures due to the mirror. The approach is based on the statistical analysis of correlated and uncorrelated emission processes, from where we derive a rough estimate of η. We thus compare the intensity of nearly 50 spectral lines recorded from the mirror-containing and the reference structure, respectively, and the results are plotted in Figure 3a. The spectrally averaged enhancement factor of 7.4_–1.3_^+1.6^ is obtained by comparing the median values for the distributions of emission intensity, which can be converted to a mean photon extraction efficiency η ≈ 6% for the device containing the metallic mirror. The 95% confidence levels for the enhancement factor are calculated according to ref (43). In the second approach, we adopt the method described by M. Gschrey et al.^44^ and directly measure η for individual QDs in mesa-processed structures. The results for η from in total six QDs (including QDs A–C) are presented in Figure 3b together with the numerically calculated values. For these mesa structures, we experimentally determine photon extraction efficiencies η above 4.4% and as high as η_B_ = 9.1% and η_C_ = 9.5% for QDs B and C, respectively. Those values demonstrate a 1 order of magnitude improvement compared to the efficiency of 0.7–0.8% estimated for the reference structure without the metallic mirror. Importantly, we obtain good agreement between the experimentally determined extraction efficiency and the theoretical prediction, for both the broadband approach (Figure 3a) and the individually investigated six QDs in mesas (Figure 3b). We attribute the small deviations between simulation and experimental values to possible nonradiative recombination channels in the QD vicinity, changes in the mesa geometry due to fabrication imperfections, and nondeterministic positioning of the QD within the mesa. These effects generally result in a lower recorded photon flux compared to the theoretical prediction, as well as in a limited probability of finding a mesa characterized by the η value as high as presented in Figure 3b in our sample. Additionally, we note that a ∼200 nm displacement of a QD from the mesa center results in an increase in the extraction efficiency as compared to a QD placed in the center. Such a displacement may explain the high η value obtained for QD B.

### Evaluation of the Photon Purity

**Figure 4:**
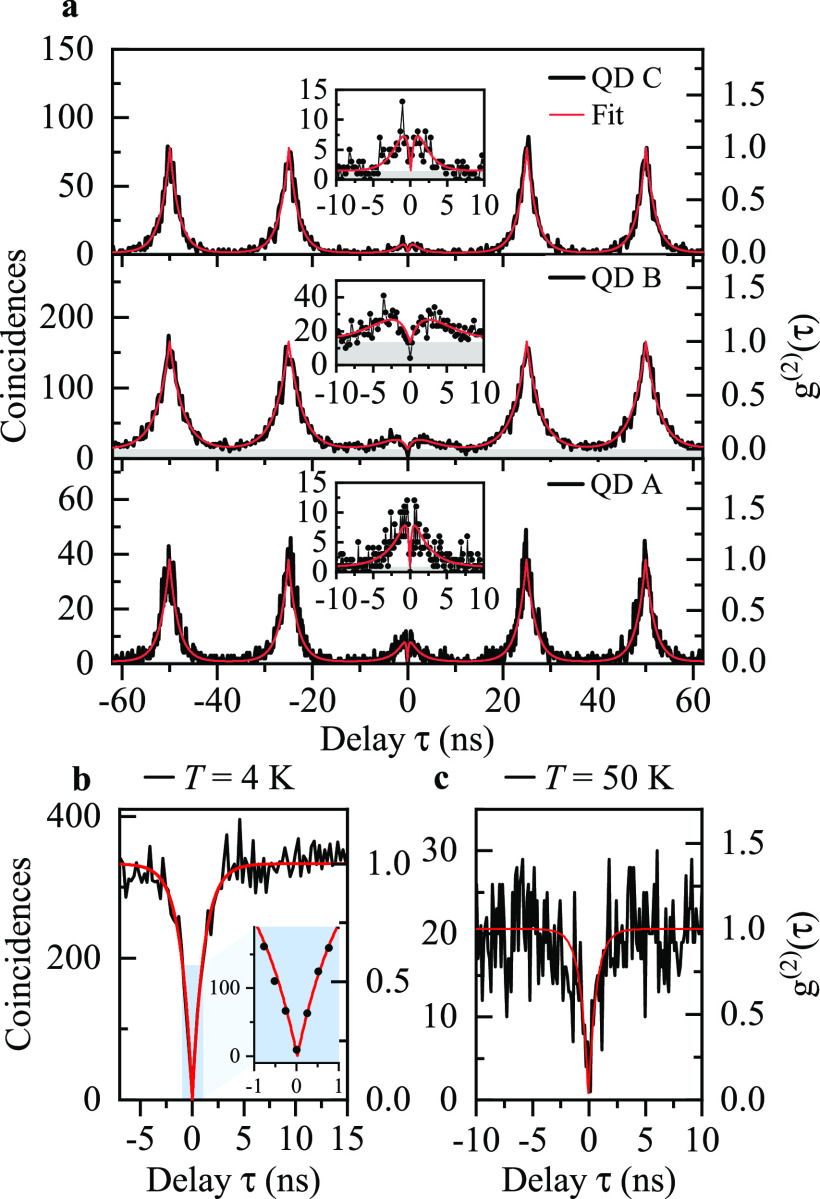
Autocorrelation histograms for CX lines. (a) Triggered single-photon emission for investigated QDs: C (top), B (center), A (bottom). Insets: Close-ups of the histograms showing coincidences around zero delay. (b, c) Single-photon emission under cw excitation for the CX in QD B, recorded (b) under the laser excitation power corresponding to the saturation of the CX μPL intensity (inset: zoom around τ = 0), and (c) at *T* = 50 K. Red lines are fits to the experimental data. Gray area in (a) shows the level of background coincidences *B* obtained by the fit with eq 1.

The order of magnitude improvement in photon extraction efficiency from InAs/InP QDs renders our structures an attractive source of single photons heterogeneously integrated with the Si platform. We evaluate in the following the quality of single-photon emission from the QDs in mesa structures by investigating the purity of single photons emitted from QDs A–C. For that purpose, we recorded the second-order correlation function *g*^(2)^(τ) exploiting off-resonant continuous wave (cw) and pulsed excitation schemes, and the obtained histograms without normalization are presented in Figure 4. For pulsed excitation (Figure 4a), we observe that the coincidences τ ≈ 0 are strongly suppressed compared to the coincidence peaks at multiples of the inverse laser repetition rate (τ_0_ = 25 ns). Furthermore, we record a significant dip in the histogram counts at short time delays |τ| < 5 ns (insets to Figure 4a). This feature indicates the capture of more than one carrier by the QD and cascaded photon emission within a single excitation, effectively resulting in multiphoton events^45^ within |τ| < 5 ns. We explain this observation with the off-resonant excitation scheme applied in our experiment, where a substantial amount of carriers are excited and trapped in the wetting layer or in other charge trap states.^45−47^ After release, those carriers are captured by the QD within the characteristic capture time τ_cap_ and produce secondary photons^48^ via exciton recombination with the time constant τ_dec_.

We fit the correlation histograms obtained with pulsed laser excitation with the function^29,49^
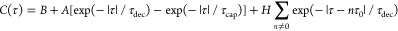
1where *B* is the level of background coincidences, *A* is a scaling parameter related to secondary photon emission, *n* ≠ 0 is the peak number, and *H* is the average height of the peaks at τ_*n*_ = *nτ*_0_. The second-order correlation function *g*^(2)^(τ) is then obtained by normalizing *C*(τ) with *H*. Evaluating *g*^(2)^(τ) at τ = 0, we obtain *g*^(2)^(0) values of 0.023 ± 0.010, 0.087 ± 0.017, and 0.018 ± 0.012 for QDs A–C, respectively. This estimation ignores coincidences produced by secondary photon emission events, which may be avoided asymptotically employing an on-resonance excitation scheme. Comparing the integrated peak area of *g*^(2)^(τ = 0) with the average peak area at τ_*n*_, as it is relevant for applications of our single-photon sources in QIP, yields values for *g*^(2)^(0)_area_ of 0.371 ± 0.020, 0.433 ± 0.018, and 0.205 ± 0.020 for QDs A–C, respectively (see the SI). Detector dark counts and uncorrelated photons also contribute to the registered histograms, where they cause a buildup of a time-independent level of coincidences *B*. We estimate this influence using eq 1 and calculate the *g*^(2)^(0)_area,cor_ values that can be associated with coincidences caused only by the QD signal. By doing so, we obtain values of *g*^(2)^(0)_area,cor_ = 0.276 ± 0.002, 0.209 ± 0.018, and 0.114 ± 0.020 for QDs A–C. The emission purity recorded in the pulsed regime is mainly limited by the capture of secondary carriers and subsequent photon emission. We note that the data are well described by our model and do not consider the capture of secondary carriers at τ_*n*_. Furthermore, with the modeling routine we determine τ_dec_ in the range 1.9–2.8 ns, which is in accordance with the CX decay time measured in time-resolved μPL (see the SI). Importantly, the obtained single-photon purity of the investigated structures is very robust for a wide range of temperatures and excitation powers. In Figure 4b,c we present correlation histograms recorded in cw excitation mode from the CX line of QD B at sample temperatures of 4 and 50 K, respectively, while optically pumping at the saturation power *P*_sat_ (see the SI for correlation histograms taken at *T* = 30 and 80 K, at 0.3 × *P*_sat_ and 0.7 × *P*_sat_, and for the summary of the obtained *g*^(2)^(0) values). We fitted the normalized histograms recorded in cw mode with a standard single-exponential function^50^ (see the SI) to extract the single-photon emission purity from the measurements. The raw data estimated *g*^(2)^(0)_raw_ value from the histogram recorded at *P*_sat_ and at *T* = 4.2 K is *g*^(2)^(0)_raw_ = 0.027_–0.027_^+0.011^ (see the SI) limited by the finite time resolution of our setup. The best fit to our data thus suggests even higher values of photon purity with *g*^(2)^(0)_fit_ = 0, with a standard error σ = 0.038, and without background correction. Such high purity of the single-photon flux in the high-excitation power regime (*P* ≥ *P*_sat_) has previously been observed only for InAs/GaAs QDs emitting at λ = (910–920 nm).^38,44^ In contrast to these sources, the structure investigated here demonstrates high brightness and close-to-ideal single-photon purity while emitting in the telecom C-band. At 50 K sample temperature, which easily can be reached with a cryogen-free Stirling cooler,^27^ a significant feature at τ = 0 is visible in the histogram, quantifying the robustness of this source of single photons. Although the emission line visibility is reduced at this temperature (see the SI for the analysis of temperature-dependent μPL of QD B), we obtain a high purity of single-photon emission with the fitted value of *g*^(2)^(0)_fit__,__50 K_ = 0.017 (σ = 0.096, without background correction; see also the SI for the histogram recorded at *T* = 80 K with *g*^(2)^(0) < 0.5).

## Conclusions

The demonstrated design of the structure with InAs/InP QDs on a metallic mirror integrated on a Si substrate paves the way toward a simplified, small-footprint, cost-effective, and scalable manufacturing process of triggered single-photon emitters operating in the telecom S-, C-, and L-bands, suitable for Si-based on-chip photonic quantum information processing. The spectral range of the InAs/InP single-photon emitters investigated here eliminates the necessity for frequency conversion to the telecom bands, potentially allowing for the implementation of distributed schemes for information processing and computation using low-loss fiber-based optical networks. Combining the robust design of our structures and the manufacturing process compatible with the existing industry standards establishes single-photon sources with high photon extraction efficiency in the broader telecom spectral range, with performance properties comparable to the DBR-based solutions but with significantly reduced fabrication-related technological demands.

The presented robust architecture, offering spectrally broad high photon extraction efficiency, is beneficial for further processing steps tailoring the photonic environment of a deterministically localized emitter. The high emitter brightness allows for its fast spatial positioning utilizing the emission imaging method successfully employed for short-wavelength (<1000 nm) QDs.^42^ However, at telecom wavelengths, the imaging relies on 2D state-of-the-art InGaAs-based matrices with yet poor efficiencies and high noise levels compared to Si-based arrays desired for shorter wavelengths. Therefore, the simplified architecture of a QD on a metal mirror can open the route toward fabrication of fully deterministic, scalable QPICs at telecom wavelengths.

## Supporting Information Available

Description of methods, extended data for optical characterization of the device, numerical simulations, and evaluation of single-photon purity (PDF)
