# Supplementary material for: Bright Quantum Dot Single-Photon Emitters at Telecom Bands Heterogeneously Integrated on Si
Source: ACS Photonics. 2022 Jun 22;9(7):2273–9. doi: 10.1021/acsphotonics.2c00027 (PMC9306001; doi:10.1021/acsphotonics.2c00027)
Supplement: Supplementary file 1 — ph2c00027_si_001.pdf [file ph2c00027_si_001.pdf]

Supplemental Material: Bright Quantum Dot Single-Photon Emitters at Telecom Bands Heterogeneously Integrated on Si

Paweł Holewa,[*,†,‡] Aurimas Sakanas,[‡] Ugur M. Gür,[¶] Paweł Mrowiński,[†] Alexander Huck,[§] Bi-Yang Wang,[‖,‡] Anna Musiał,[†] Kresten Yvind,[‡,⊥] Niels Gregersen,[‡] Marcin Syperek,[*,†] and Elizaveta Semenova[*,‡,⊥]

†*Laboratory for Optical Spectroscopy of Nanostructures, Faculty of Fundamental Problems of Technology, Department of Experimental Physics, Wrocław University of Science and Technology, Wyb. Wyspiańskiego 27, 50-370 Wrocław, Poland*

‡*DTU Fotonik, Technical University of Denmark, Kongens Lyngby 2800, Denmark*

¶*DTU Electrical Engineering, Technical University of Denmark, 2800 Kongens Lyngby, Denmark*

§*Center for Macroscopic Quantum States (bigQ), Department of Physics, Technical University of Denmark, 2800 Kongens Lyngby, Denmark*

‖*Hefei National Laboratory for Physical Sciences at Microscale, University of Science and Technology of China, Hefei, Anhui 230026, China*

⊥*NanoPhoton-Center for Nanophotonics, Technical University of Denmark, 2800 Kongens Lyngby, Denmark*

E-mail: pawel.holewa@pwr.edu.pl; marcin.syperek@pwr.edu.pl; esem@fotonik.dtu.dk

Number of pages in this file: 21, number of figures: 7, number of tables: 4.

Contents

Methods **S-2**

QD fabrication S-2

QD integration on Si substrate S-3

Optical experiments S-4

Determination of the photon extraction efficiency S-4

Determining the single-photon purity S-6

Numerical calculations S-7

Identification of excitonic complexes **S-7**

FDTD calculations **S-10**

Time-resolved microphotoluminescence for CX lines in QDs A-C **S-11**

CW autocorrelation histograms **S-12**

Temperature-dependent photoluminescence of QD B **S-14**

Summary of derived parameters **S-16**

Methods

QD fabrication

The QDs are grown in the low-pressure MOVPE TurboDisc® reactor using arsine (AsH_3), phosphine (PH_3), tertiarybutylphosphine (TBP) and trimethylindium (TMIn) precursors with H_2 as a carrier gas. The growth sequence starts with the deposition of a 0.5 µm-thick

InP buffer layer on a (001)-oriented InP substrate at 610 °C subsequently epitaxially covered by a 200 nm-thick $In_{0.53}Ga_{0.47}As$ sacrificial layer lattice-matched to InP and a 244 nm-thick InP layer. Then, the temperature is decreased to 483 °C, stabilized under TBP for 180 s and AsH_3 for 27 s. Finally, nucleation of QDs occurs in the Stranski-Krastanov growth mode after deposition of nominally 0.93 mono-layer thick InAs under TMIn and AsH_3 flow rates of 11.8 µmol/min and 52.2 µmol/min, respectively. Nucleated QDs are annealed for 60 s at the growth temperature in AsH_3 ambient, before the temperature is increased for 30 s to 515 °C and the annealing continues for another 30 s. Deposition of a 244 nm-thick InP capping layer (12 nm at 515 °C, and the remaining 232 nm after increasing the temperature up to 610 °C) finishes the growth sequence.

The reference structure contains the same InAs/InP quantum dots without InGaAs sacrificial layer and metallic mirror (no processing was done on this structure).

QD integration on Si substrate

To integrate the QD structure on Si, a 100 nm-thick layer of SiO_2 is deposited on top of the InP-based structure using plasma-enhanced chemical vapor deposition (PECVD) with the rate of 0.99 nm/s, and subsequently covered by a 100 nm-thick Al layer deposited via electron-beam evaporation with the rate of 90 Å/s. After flipping the structure bottom-up, it is bonded to the Si substrate. The bonding procedure includes, first, spin coating of the AP3000 adhesion promoter and benzocyclobutene (BCB) on Si and AP3000 on the InP wafer, and second, both structures are bonded at 250 °C in vacuum under an applied force of $\sim$ 2 kN. Plasma ashing disposes superfluous BCB from the back-side of the InP wafer. Afterward, the InP substrate is removed in HCl and the InGaAs etch stop layer in H_2SO_4:H_2O_2:H_2O=1:8:80 mixture. For the sample with mesas, HSQ resist (a high purity silsesquioxane-based semiconductor grade polymer) is spin-coated and exposed using electron-beam lithography and developed in water-diluted AZ400K. The mesa pattern is then transferred to the InP by inductively coupled plasma-reactive ion etching (ICP-RIE)

followed by HSQ removal in a buffered oxide etch (BHF). The mesa height is 300 nm as measured with an atomic force microscope.

Optical experiments

For the optical experiments, the structure is hold in a helium-flow cryostat allowing for controlled sample temperatures in the range of 4.2 K to 300 K. For our standard μPL studies, the structures are optically excited through a high numerical aperture (NA = 0.4) microscope objective with 20× magnification with 660 nm or 787 nm light generated with semiconductor laser diodes, respectively. The same objective is used to collect the PL and direct it for spectral analysis to a 1 m-focal-length monochromator equipped with a liquid-nitrogen-cooled InGaAs multichannel array detector, providing spatial and spectral resolution of ≈ 2 μm and ≈ 25 μeV, respectively. Polarization properties of emitted light are analyzed by rotating a half-wave plate mounted before a fixed high-contrast-ratio (10^6:1) linear polarizer, both placed in front of the monochromator entrance.

Autocorrelation histograms, photon extraction efficiency, and TRPL are measured in a similar setup. In this setup, the structures are excited by a train of ∼ 50 ps-long pulses with a repetition frequency of 40 MHz or 80 MHz, and the central photon wavelength of 805 nm. The collected photons are dispersed by a 0.32 m-focal-length monochromator equipped either with a InGaAs multichannel array detector or NbN-based superconducting nanowire single-photon detectors (SNSPDs, Scontel) with ∼85 % quantum efficiency in the 1.5 μm to 1.6 μm range and ∼ 200 dark counts per second. A multichannel picosecond event timer (PicoHarp 300 by PicoQuant) analyzes the single photon counts as time-to-amplitude converter with 256 ps channel time bin width. The overall time resolution of the setup is ∼80 ps.

Determination of the photon extraction efficiency

To determine the value of photon extraction efficiency η_{QD}, we follow the method described in Ref.[1] First, we estimate the efficiency of the setup η_{Setup} by reflecting a laser tuned to the

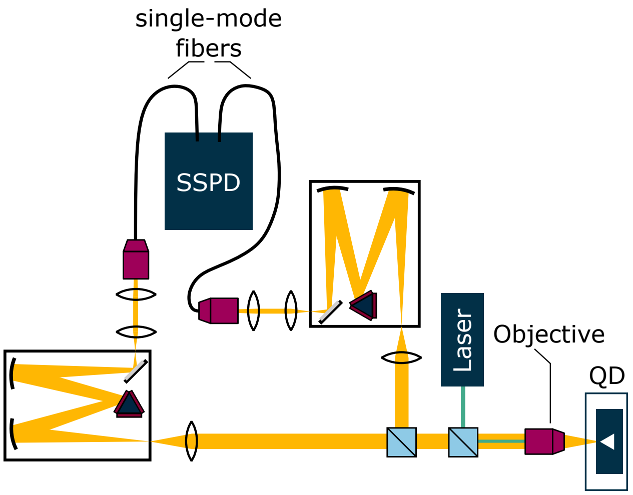

Figure S1: Scheme of the experimental setup used for correlation spectroscopy and determination of the extraction efficiency.

investigated QD emission range off a silver mirror placed in the setup instead of the structure. The laser beam is attenuated with neutral density filters to achieve the SNSPD count rate in the MHz range. This number is corrected by the measured mirror reflectivity, attenuation of filters, transmission of the cryostat window and the microscope objective. Based on the laser power coming on the silver mirror, the estimated setup efficiency is $\eta_{\text{Setup}} = (0.18 \pm 0.06)\,\%$, with the uncertainty being the standard deviation $\sigma(\eta)$ of $\eta_{\text{Setup}}(\lambda)$, mainly steming from the slight discrepancies in the fiber in-coupling efficiencies for different wavelengths. We performed the extraction efficiency experiments in the correlation spectroscopy setup, presented in Fig. S1. The signal emitted from the sample passes through the following elements of the setup on the way to the detector: cryostat window (spectrally averaged transmission $T \approx 90\,\%$), microscope objective ($T \approx 55\,\%$), two beam splitters (introducing the optical excitation and splitting the QD signal, $T \approx 40\,\%$ each), monochromator ($T \approx 15\,\%$), fiber in-coupling (efficiency $\sim$18 %) and SNSPD (efficiency $\sim$85 %). The multiplication of these values leads to the same η_{Setup} as determined using the described method, adopted from Ref.[1]

Then, we excite the QDs non-resonantly with a pulsed laser diode with $f_{\text{rep}} = 80\,\text{MHz}$ repetition rate at the saturation power for each QD. We collect the emission with the mi-

croscope objective (NA $= 0.4$), sum the SNSPD count rates for CX and X lines (n_{QD}), as only one excitonic complex can radiatively decay at a time, and correct them by η_{Setup}. Taking into account the laser repetition f, we estimate the photon extraction efficiency $\eta_{\mathrm{QD}} = n_{\mathrm{QD}}/(f \times \eta_{\mathrm{Setup}})$. The error bars for photon extraction efficiencies are calculated by propagating the $\sigma(\eta)$ uncertainty. This method assumes unity internal quantum efficiency of QDs ($\eta_{\mathrm{int}} = 100\,\%$), respectively the QD photon repetition rate equals f_{rep}. It is however difficult to determine experimentally the contribution of non-radiative recombination and hence the real value of η_{int}. The assumption of $\eta_{\mathrm{int}} = 100\,\%$ thus determines a lower limit of η_{QD} due to a possible overestimation of the total number of photons emitted by the QD (n_{QD}). Finally, we correct the measured η values for the QDs A-C by the factor $\sqrt{1 - g^{(2)}(0)_{\mathrm{area}}}$ to account for the secondary photons due to the refilling of QD states that contribute to the measured photon flux.[2,3] This procedure only slightly reduces the η values by 16 %, 11 %, and 5 % for QDs A, B, and C, respectively. With this correction, the highest η values are 9.5 % and 9.1 % for QDs C and B, respectively.

Determining the single-photon purity

For the pulsed QD excitation, we calculate the $g^{(2)}(0)$ value including the histogram background contribution B:

$$g^{(2)}(0)_{\mathrm{area}} = \int_{-T_0/2}^{T_0/2} \left[B + A\left[\exp\left(-|\tau|/\tau_{\mathrm{dec}}\right) - \exp\left(-|\tau|/\tau_{\mathrm{cap}}\right)\right]\right] \mathrm{d}\tau \Big/ \int_{-T_0/2}^{T_0/2} \left[B + H \exp\left(-|\tau|/\tau_{\mathrm{dec}}\right)\right] \mathrm{d}\tau, \tag{S1}$$

and with the background contribution subtracted:

$$g^{(2)}(0)_{\mathrm{area}} = \int_{-T_0/2}^{T_0/2} A\left[\exp\left(-|\tau|/\tau_{\mathrm{dec}}\right) - \exp\left(-|\tau|/\tau_{\mathrm{cap}}\right)\right] \mathrm{d}\tau \Big/ \int_{-T_0/2}^{T_0/2} H \exp\left(-|\tau|/\tau_{\mathrm{dec}}\right) \mathrm{d}\tau. \tag{S2}$$

For the histograms recorded in cw mode we use the standard equation

$$C(\tau) = N\left[1 - \left(1 - g^{(2)}_{\text{fit}}(0)\right)\exp\left(-|\tau|/t_{\text{r}}\right)\right], \tag{S3}$$

where N is the average coincidence level at $|\tau| \gg 0$. The purity is extracted as $\mathcal{P} = 1 - g^{(2)}(0)$, in particular, for the raw-data estimated purity $\mathcal{P}_{\text{raw}} = C(0)/N$.

Numerical calculations

The structure is modeled with a modal method (MM) employing a true open geometry boundary condition.[4] Here, the geometry is divided into uniform layers along a propagation z axis, and the field is expanded on eigenmodes of each uniform layer. The eigenmode expansion coefficients in the QD layer are computed using the reciprocity theorem,[5] and the fields are connected at each layer interface using the S matrix formalism.[5,6] The far field is then determined using the field equivalence principle and radiation integrals.[7] The extraction efficiency is defined as $\eta = P_{\text{lens, NA}}/P_{\text{in}}$, where $P_{\text{lens, NA}}$ is the power detected by the lens with NA = 0.4 in the far field, and P_{in} is the total power emitted from the dipole.

Identification of excitonic complexes

To demonstrate the optical properties of the QDs, three exemplary emitters representing the L- (QD A), C- (QD B), and S- (QD C) telecom bands are chosen, with their µPL spectra presented in Figs. 2b-2d. The excitonic complexes are identified based on the excitation power-dependent µPL intensity $I_{\mu\text{PL}}$ [shown in Figs. S2a-S2c], and polarization-resolved µPL investigations [results shown in Figs. S2d-S2e]. We obtain the expected[8,9] linear, superlinear and almost quadratic power dependences for excitons, trions, and biexcitons, respectively, with the following exponents b from fitting the power dependence $I_{\mu\text{PL}} = aP^b$ to the line intensities: $b_{\text{X}} = 0.98 \pm 0.08$, $b_{\text{XX}} = 1.77 \pm 0.11$, $b_{\text{CX}} = 1.04 \pm 0.04$ (QD A), $b_{\text{X}} = 1.00 \pm 0.15$, $b_{\text{XX}} = 1.75 \pm 0.15$, $b_{\text{CX}} = 1.12 \pm 0.18$ (QD B), and $b_{\text{X}} = 1.02 \pm 0.02$, $b_{\text{XX}} = 1.67 \pm 0.10$,

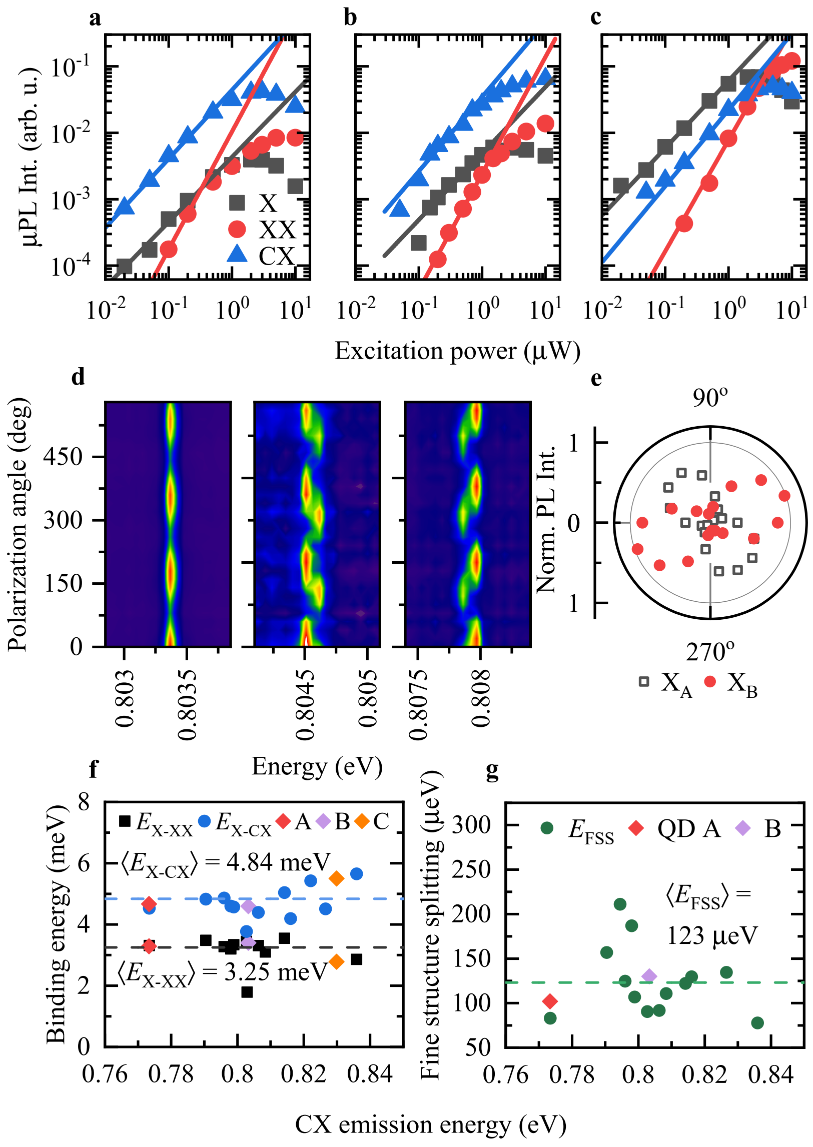

Figure S2: Excitonic complexes in InAs/InP QDs. **a-c**, Excitation power-dependent μPL intensity of identified lines in QDs: **a** A, **b** B, **c** C. **d**, Polarization-resolved μPL signal for excitonic complexes in QD B. **e**, Normalized polarization-resolved μPL intensity for X and XX in QD B. **f**, Binding energies for trions ($E_{\mathrm{X-CX}}$) and for biexcitons ($E_{\mathrm{X-XX}}$), with values for QDs A, B, and C marked with diamonds. **g**, Exciton fine structure splitting (E_{FSS}).

$b_{\mathrm{CX}} = 1.15 \pm 0.05$ (QD C), where b_{X}, b_{XX}, b_{CX} are exponents for the X, XX, and CX lines, respectively.

An exemplary polarization-resolved μPL map is shown in Fig. S2d with the traces that help to unambiguously ascribe the lines, with X and XX oscillating in anti-phase (right and center panel, respectively), revealing the exciton fine structure splitting with the energy

$E_{\mathrm{FSS}} = 91\,\mu\mathrm{eV}$. The slight non-orthogonality of X states, visible in the intensities of both bright exciton (X_A and X_B) and biexciton (XX_A and XX_B) states (Fig. S2e), as well as CX intensity modulation, evidences the valence-band mixing between heavy- and light-hole states due to in-plane QD shape asymmetry and anisotropic strain effects[10–12] what has also been observed for trions in similar InP-based QDs.[13] Based on the polarization dependence of the X line µPL intensity, we determine the degree of linear polarization $\mathrm{DOLP_X} = (42.8 \pm 3.2)$ % and the amplitude of the hole states mixing[12] $\beta_X = (36.3 \pm 2.7)$ %. For the CX, we observe the lack of the emission energy dependence on the linear polarization angle (Fig. S2d, left panel), as expected for the trion spin-singlet state, and based on its intensity we calculate the parameters $\mathrm{DOLP_{CX}} = (36.4 \pm 3.2)$ % and $\beta_{\mathrm{CX}} = (31.1 \pm 2.7)$ %.

We perform in an similar manner the identification of complexes for other QDs in the investigated structures. Based on polarization-resolved and excitation power-dependent µPL spectra of InAs/InP QDs in the structure with mirror which emit in the range of 1.48 µm to 1.6 µm, we determine the binding energies for trions ($E_{\mathrm{X-CX}}$) and for biexcitons ($E_{\mathrm{X-XX}}$). The summary of determined binding energies for biexcitons and trions, and the E_{FSS} values are shown in Figs. S2f-S2g. We find that the distributions of biexciton and trion binding energies are narrow and the values for QDs A-C are close to their typical values (see the diamonds marking values for QDs A, B, and C). We calculate the average values of $\langle E_{\mathrm{X-CX}} \rangle = 4.84\,\mathrm{meV}$ and $\langle E_{\mathrm{X-XX}} \rangle = 3.25\,\mathrm{meV}$. The obtained values are spectrally-independent and only slightly spread around the average values, therefore they help in the identification of excitonic complexes in the investigated QDs A-C. For the fine structure splitting energy, we calculate the average value of $\langle E_{\mathrm{FSS}} \rangle = 123\,\mu\mathrm{eV}$.

Finally, for QD C we present the cross-correlation between XX-X and CX-X lines to unambiguously prove the identification of excitonic lines and the fact that they origin from the same QD. Here, X emission events are registered by the stopping detector. The histograms are shown in Fig. S2g for XX-X and Fig. S2h for CX-X cross-correlation measurements. In the case of XX-X, we find a strong bunching for positive delays, evidencing the cascaded

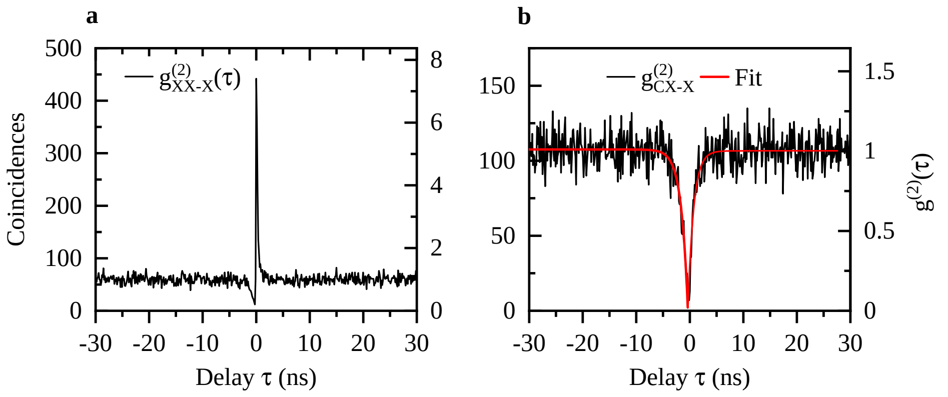

Figure S3: **a**, Cross-correlation of XX and X lines of QD C. **b**, Cross-correlation of CX and X lines of QD C with fit line (red).

XX-X emission, while for the CX-X case we see an asymmetric dip which we fit with the function of the form $g^{(2)}_{\mathrm{CX-X}}(\tau) = A\,[1 - \exp{(\tau/t_{\mathrm{r}})}]$ separately for $\tau < 0$ and $\tau > 0$, where A is a scaling factor, τ is the time delay, and t_{r} is the antibunching time constant. We find that the t_{r} is different for positive and negative delays: $t_{\mathrm{r}} = 1.25 \pm 0.08$ ns and $t_{\mathrm{r}} = 1.06 \pm 0.08$ ns, respectively, corresponding with the CX and X emission.

FDTD calculations

In this section we investigate the influence of the QD position displacement in the mesa structure on the extraction efficiency. For that purpose, we employ finite-difference time-domain (FDTD) 3D Electromagnetic Simulator provided by Lumerical Inc.,[14] as a complementary tool to the previous one based on the modal method (MM).[4] More details of the employed FDTD method can be found elsewhere.[15] In order to establish convergence between the two numerical methods we first compare the results for the identical photonic mesa structures with D = 2 µm containing a point dipole at the central position $\Delta x = 0$ nm. Then, the FDTD approach was tuned to minimize deviation with respect to the modal method by slight change of the numerical aperture of the collected emission or the position of the 2D field-power monitor located above the mesa structure. Such tuning mechanism is visualized in Fig. S4a as the black shaded area which overall is qualitatively similar to the results

obtained by the modal method.

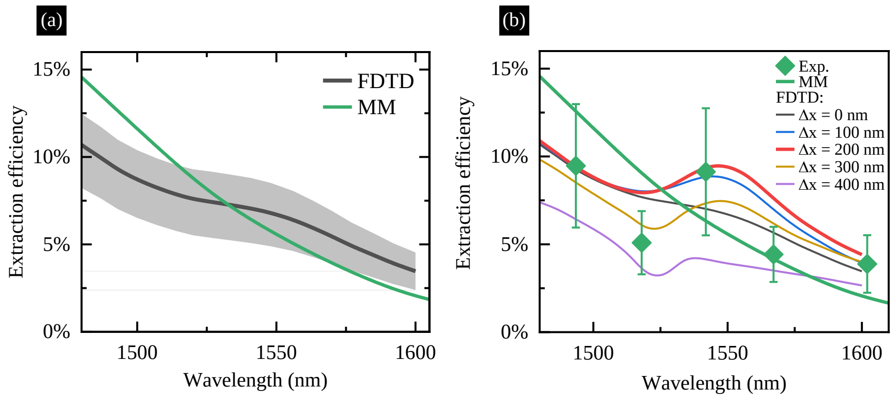

Figure S4: Analysis of the influence of the QD position in the mesa on the extraction efficiency. **a**, Comparison of calculated extraction efficiency between the FDTD and MM methods for the QD in the center of the mesa ($\Delta x = 0$ nm). The shaded uncertainty range results from different vertical screen positions (0.8 µm to 2.4 µm above the mesa surface) **b**, Dispersion of the extraction efficiency for different QD displacement from the center of the mesa (Δx).

Next, such consistent FDTD model was used to calculate the dispersion of extraction efficiency for a variety of the dipole displacements in the range of $\Delta x = 0$ nm to 400 nm. Starting from $\Delta x = 100$ nm, a local enhancement of extraction efficiency around 1.55 µm is already observable and this effect is further strengthened for $\Delta x = 200$ nm displacement, as it is shown in Fig. S4b. Further shift of the dipole position causes decrease of the extraction efficiency values, and taking into account the experimental results we observe the best match for QD B at $\Delta x = 200$ nm, suggesting that the QDs are placed in between $\Delta x \approx 0$ nm to 200 nm.

Time-resolved microphotoluminescence for CX lines in QDs A-C

Low-temperature ($T = 4.2$ K) time-resolved µPL (TRPL) traces registered for QDs A, B, and C are presented in Fig. S5 with solid black lines. Each trace is best fitted with a single-

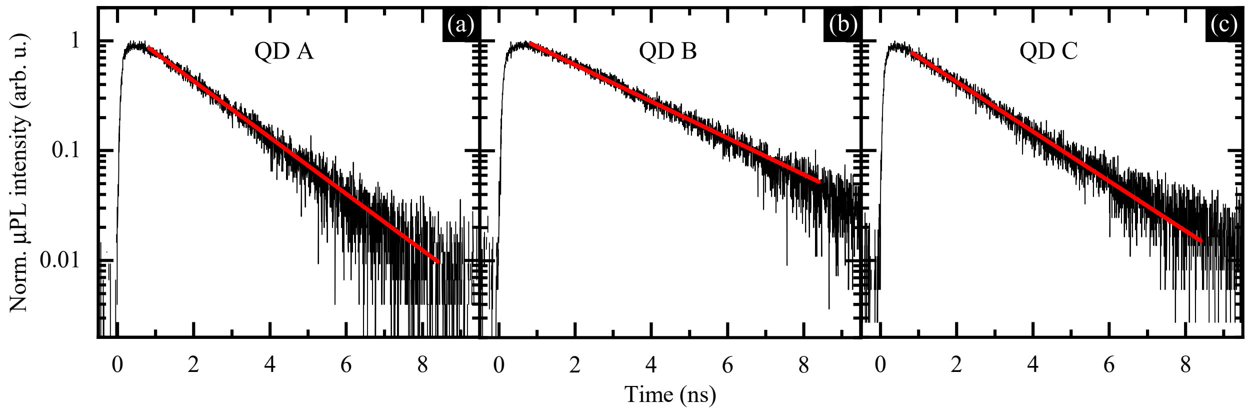

Figure S5: Time-resolved μPL traces for trions in QDs A-C: **a**, QD A, **b**, QD B, **c**, QD C. Red solid lines are fit lines to the experimental data (black lines), according to Eq. (S4).

exponential decay function (red solid lines) to extract decay time constant τ_{PL}. We use the function of the form:

$$I(t) = A \exp\left(-t/\tau_{\mathrm{PL}}\right), \tag{S4}$$

where $I(t)$ is the TRPL intensity at time t, and A is the amplitude of the signal. The extracted τ_{PL} values are 1.69 ± 0.01 ns (QD A), 2.61 ± 0.01 ns (QD B), and 1.92 ± 0.01 ns (QD C). The τ_{PL} times are rather typical for single InAs/InP QDs, independently of their exact size and symmetry[16–19] and agree with the τ_{dec} values (see comparison in Tab. S2).

CW autocorrelation histograms

In this section we present the histograms that broaden the discussion of the single-photon emission quality for investigated SPEs, presented in Fig. 4 in the article. Here, we focus on the cw excitation of the CX lines, and we show the autocorrelation histograms for QDs A and C in Fig. S6a and Fig. S6b, respectively, together with fit lines. The histograms were recorded for the laser excitation power corresponding to the $0.7 \times P_{\mathrm{Sat}}$ of the respective lines. We observe an additional weak bunching effect which we attribute to the blinking caused by the interaction with carrier traps in the QD vicinity or background doping,[3,20] and we fit

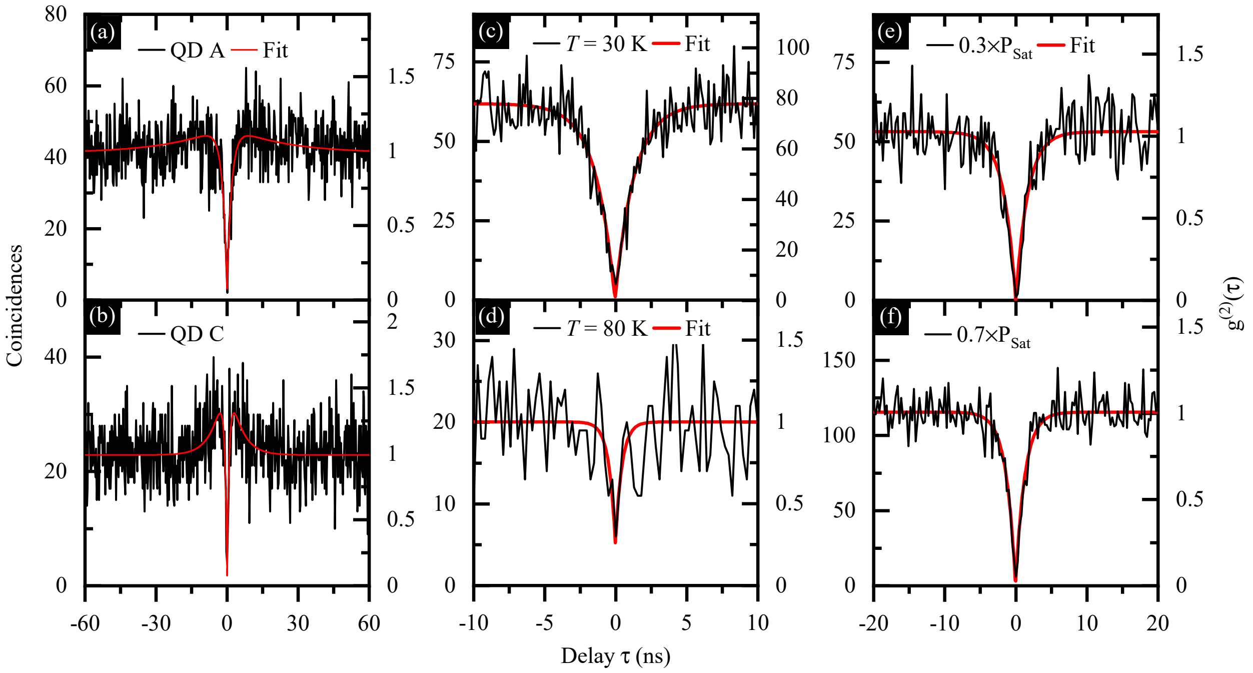

Figure S6: Evaluation of the single-photon emission purity for CX lines under cw excitation in QD **a**, A and **b**, C, and for CX line in QD B at elevated temperatures: recorded and C QD B, at **c**, $T = 30\,\mathrm{K}$, **d**, $T = 80\,\mathrm{K}$, and for **e**, $0.3 \times P_{\mathrm{Sat}}$ and **f**, $0.7 \times P_{\mathrm{Sat}}$.

the normalized histograms with the function[21]

$$g^{(2)}(\tau) = 1 - A\exp\left(-|\tau|/\tau_1\right) + B\exp\left(-|\tau|/\tau_2\right), \tag{S5}$$

where A and B are fit parameters, while τ_1 and τ_2 are antibunching and bunching time constants, respectively. The $g^{(2)}(0)$ value is obtained as $g^{(2)}(0) = 1 - A + B$. Determined parameters are $g^{(2)}(0) = 0.074$ ($\sigma = 0.062$), $\tau_1 = 1.93 \pm 0.15$ ns, and $\tau_2 = 22.7 \pm 3.0$ ns for QD A, and $g^{(2)}(0) = 0.07$ ($\sigma = 0.11$), $\tau_1 = 0.97 \pm 0.09$ ns, and $\tau_2 = 4.81 \pm 0.45$ ns for QD C. These $g^{(2)}(0)$ values are displayed in Tab. S3 for their easier comparison.

Then, we show the histograms for CX in QD B registered under the same excitation conditions as in Figs. 4b-c, except for the temperature of the structure (Figs. S6c-S6d) and excitation power (Figs. S6e-S6f). We fit the normalized histograms with the standard function shown in Eq. (S3). For the Stirling-compatible temperature of $T = 30\,\mathrm{K}$ we obtain almost perfect single-photon emission with the purity of $g^{(2)}(0)_{30\,\mathrm{K}} = 0$ ($\sigma = 0.054$, Fig. S6c).

Moreover, we record the histogram at 80 K sample temperature, achievable with a liquid nitrogen dewar. Although the reduction of the signal-to-noise ratio for investigated line results in a pronounced noise in the recorded histogram, the dip at zero delay is still visible and the single photon purity can be estimated. We obtain $g^{(2)}(0)_{\text{fit},80\,\text{K}} = 0.25(\sigma = 0.19)$. We note that the achieved purity at $T = 80\,\text{K}$ is higher than the record values of $g^{(2)}(0) = 0.34$ and $g^{(2)}(0) = 0.33$ previously reported at this temperature and at 1.55 µm emission wavelength achieved with InAs/InAlGaAs/InP quantum dashes[22,23] and InAs/GaAs QDs grown with the metamorphic approach,[24] respectively. Importantly, both these previous approaches are obtained in different carrier confinement conditions, InAlGaAs or GaAs barriers respectively, and thus cannot be directly compared with the pure InAs/InP system investigated here.

Next, for the C-band QD B we study the quantum nature of the emission in the excitation power-dependent photon autocorrelation measurements under cw non-resonant excitation. Figs. S6e-S6f present the autocorrelation histograms corresponding to the excitation of the CX line under $0.3\times P_{\text{Sat}}$ (Fig. S6e), and $0.7\times P_{\text{Sat}}$ (Fig. S6f), where P_{Sat} is the laser excitation power corresponding to the saturation of the line's µPL intensity. We fit the histograms using Eq. (S3), and achieve the values of $g^{(2)}(0) = 0$ for all probed excitation powers, with $\sigma = 0.075$, 0.056, 0.038 for $0.3 \times P_{\text{Sat}}$, $0.7 \times P_{\text{Sat}}$, and P_{Sat}[1], respectively. The increasing pump rate W_p results in the decrease of t_{r} so that $t_{\text{r}} = (1.70 \pm 0.19)$ ns, $t_{\text{r}} = (1.31 \pm 0.11)$ ns, and $t_{\text{r}} = (1.12 \pm 0.06)$ ns for $0.3 \times P_{\text{Sat}}$, $0.7 \times P_{\text{Sat}}$, and P_{Sat}, respectively.

The obtained $g^{(2)}(0)$ for CX in the QD B are repeated in Tab. S4.

Temperature-dependent photoluminescence of QD B

The temperature-dependent µPL of the QD B is recorded in the temperature range of $T = 5\,\text{K}$ to $120\,\text{K}$ and analyzed before recording the autocorrelation histograms at elevated temperatures, shown in Fig. 4c. The spectra, the temperature-dependent quench of CX and X lines, and the linewidth broadening of the CX line are presented in Fig. S7.

[1]The histogram recorded for P_{Sat} is shown in Fig. 4b

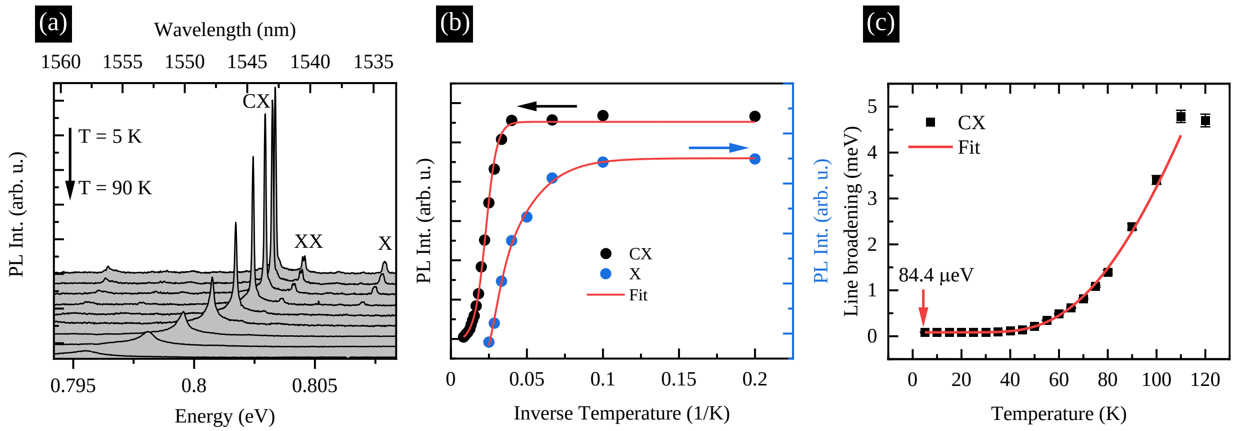

Figure S7: Analysis of the temperature-dependent μPL spectra for QD B. **a**, μPL spectra taken at different temperatures in the range of $T = 5\,\mathrm{K}$ to $90\,\mathrm{K}$. **b**, μPL intensity for X and CX lines fitted with the Arrhenius formula (red lines), according to Eq. (S6). **c**, Broadening of the CX line with the fitted temperature dependence of Eq. (S7).

To identify the most efficient carrier excitation channels, the temperature-dependent μPL intensity is fitted with a standard formula assuming two activation processes: [25]

$$I\left(T\right) = \frac{I_0}{1 + B_1 \exp\left(-E_{\mathrm{a},1}/k_{\mathrm{B}}T\right) + B_2 \exp\left(-E_{\mathrm{a},2}/k_{\mathrm{B}}T\right)}, \tag{S6}$$

where I_0 is the PL intensity for $T \to 0$, $E_{\mathrm{a},1}$ and $E_{\mathrm{a},2}$ are activation energies, and B_1 and B_2 are relative rates corresponding to the efficiency of involved processes. We achieve the activation energies of $E_{\mathrm{a},1} = 23.6 \pm 0.8$ meV and $E_{\mathrm{a},2} = 81.1 \pm 15$ meV for CX and $E_{\mathrm{a},1} = 5.0 \pm 1.5$ meV and $E_{\mathrm{a},2} = 22.2 \pm 7.3$ meV for X. We find that for both lines the dominant process responsible for the μPL intensity quench is the one corresponding to the $E_{\mathrm{a},2}$ energy, as the rates ratio $B_1 : B_2$ is $1 : 264$ and $1 : 402$ for CX and X lines, respectively. Interestingly, both complexes share the activation energy in the range of $\sim$22 meV to 24 meV, in agreement with the value obtained for the CX line in similar QDs,[13] where the values of $E_{\mathrm{a},1} = 0.8 \pm 0.5$meV and $E_{\mathrm{a},2} = 23 \pm 5$meV were reported for a CX. There, the process corresponding to the $E_{\mathrm{a},2}$ activation energy was attributed as the charge transfer to higher orbital states, based on the band structure calculations within the 8-band $k \cdot p$ framework. Furthermore, for a QD emitting around the 1.55 μm spectral range, the calculated energy

distance for holes between their QD and WL ground states is in the range of 70 meV to 90 meV,[13] therefore we attribute the $E_{a,2} = 81.1 \pm 15$meV estimated for the CX as the excitation of the hole from the QD B to the WL ground state.

The temperature dependence of linewidth was fitted with the formula that includes the contribution of thermally-activated phonon sidebands to the zero-phonon line,[26,27]

$$\Gamma(T) = \Gamma\,(4.2\,\mathrm{K}) + a\left[\exp\left(\frac{E_{\mathrm{ph},\Gamma}}{k_{\mathrm{B}}T}\right) - 1\right]^{-1}, \tag{S7}$$

where k_{B} is the Boltzmann constant, parameter $a = 72.8 \pm 6.8$ meV and $E_{\mathrm{ph},\Gamma} = 27.39 \pm 0.55$ meV is an average energy of phonons. From the fitting we obtain the initial CX line broadening of $\Gamma\,(4.2\,\mathrm{K}) = 84.4 \pm 1.2$ µeV what is above the lifetime-limited linewidth (0.5 µeV to 0.8 µeV, according to TRPL data in Fig. S5) as well as above the spectral resolution of the µPL setup ($\sim$25 µeV) and so points to the presence of spectral diffusion due to the deep charge traps in the QD vicinity, also on the etched mesa walls.[28] On the other hand, the initial broadening is much lower than in the previously fabricated structure with InAs/InP QDs in mesas of 340 µeV.[13]

Summary of derived parameters

In Tab. S1 we provide the fit parameters for the histograms obtained under cw laser excitation for QDs A, B, and C. The purity $\mathcal{P}$ is defined as $\mathcal{P} = 1 - g^{(2)}(0)$. For the purity determination we take the fitted values of $g^{(2)}(0)_{\mathrm{fit}}$ unless $g^{(2)}(0)_{\mathrm{fit}} = 0$. In such cases, we employ the more conservative estimation of purity, utilizing the $g^{(2)}(0)_{\mathrm{raw}} = C(0)/N$ value (Eq. (S3)).

In Tab. S2 we give the fit parameters for histograms obtained for the pulsed laser excitation (see Methods section for the fitting formula). B is the level of background coincidences, A is a scaling parameter related to secondary photon emission, H is an average non-zero peak height, τ_{dec} and τ_{cap} are the decay and capture time constants, respectively. The corresponding histograms are shown in Fig. 4 and Figs. S6a-S6b. We give also the µPL decay

Table S1: Fit parameters of single-photon emission under cw excitation (Figs. S6a-S6b for QDs A and C, Fig. S6f for QD B).

Different QDs, cw excitation			
QD	$g^{(2)}(0)_{\text{fit}}$	$g^{(2)}(0)_{\text{raw}}$	$\mathcal{P}$
A	0 $(\sigma = 0.11)$	0.0480	$\left(95.2^{+4.8}_{-6.2}\right)$ %
B	0 $(\sigma = 0.056)^{*}$	0.0519	$\left(94.8^{+5.2}_{-0.4}\right)$ %
C	0.07 $(\sigma = 0.11)$	0.173	$\left(93^{+7}_{-11}\right)$ %

* At $0.7 \times P_{\text{Sat}}$.

time τ_{PL} recorded in time-resolved μPL experiment (the corresponding μPL decay traces are presented in Fig. S5). The uncertainties given in Tab. S2 are standard errors of the fitting procedure (σ).

Table S2: Fit parameters of single-photon emission under CW and pulsed excitation for QDs A-C (Fig. 4a) with PL decay times (Fig. S5).

	Different QDs, pulsed excitation					TRPL
QD	B	A	H	τ_{cap}	τ_{dec}	τ_{PL}
A	0.86 ± 0.05	12.49 ± 0.90	37.30 ± 0.16	0.34 ± 0.07 ns	1.91 ± 0.02 ns	1.69 ± 0.01 ns
B	13.32 ± 0.16	176 ± 15	152.35 ± 0.47	2.28 ± 0.05 ns	2.80 ± 0.02 ns	2.61 ± 0.01 ns
C	1.41 ± 0.15	11.2 ± 2.0	76.82 ± 0.58	0.44 ± 0.17 ns	1.99 ± 0.02 ns	1.92 ± 0.01 ns

* At $0.7 \times P_{\text{Sat}}$.

Tab. S3 gives the derived $g^{(2)}(0)$ function values for pulsed excitation together with corresponding purity. We give both the $g^{(2)}(0)$ value based on the level of coincidences at $\tau = 0$ compared with H, and based on the area under the zero histogram peak. See Methods section for the applied formulas with and without the background correction. The uncertainties given in Tab. S3 are combined standard uncertainties based on A and H standard fitting errors.

Tab. S4 displays the fit parameters obtained for CX in QD B, for the excitation-power- and temperature-dependent autocorrelation histograms. Again, for the determination of $\mathcal{P}$ we use the fitted values of $g^{(2)}(0)_{\text{fit}}$ unless $g^{(2)}(0)_{\text{fit}} = 0$. In such cases, we utilize the $g^{(2)}(0)_{\text{raw}} = C(0)/N$ value.

Table S3: $g^{(2)}(0)$ values and corresponding purity $\mathcal{P}$ of the single-photon emission under pulsed excitation (Fig. 4a) derived based on the fit parameters given in Tab. S2.

Different QDs, pulsed excitation						
	Function value approach		Peak area approach			
	Fit value at $\tau = 0$		Histogram background included		Histogram background subtracted	
QD	$g^{(2)}(0) = B/H$	$\mathcal{P} = 1 - g^{(2)}(0)$	$g^{(2)}(0)_{\text{area}}$	$\mathcal{P}_{\text{area}}$	$g^{(2)}(0)_{\text{area}}$	$\mathcal{P}_{\text{area}}$
A	0.023 ± 0.010	97.7 ± 1.0 %	0.371 ± 0.020	62.9 ± 2.0 %	0.276 ± 0.002	72.4 ± 2.0 %
B	0.087 ± 0.017	91.3 ± 1.7 %	0.433 ± 0.018	56.7 ± 1.8 %	0.209 ± 0.018	79.1 ± 1.8 %
C	0.018 ± 0.012	98.2 ± 1.2 %	0.205 ± 0.02	79.5 ± 2.0 %	0.114 ± 0.02	88.6 ± 2.0 %

* At $0.7 \times P_{\text{Sat}}$.

Table S4: The histogram fit parameters obtained for CX in QD B, for excitation power- and temperature-dependent autocorrelation histograms [see Figs. 4b-c and Figs. S6c-S6f].

QD B, cw excitation							
Excitation power series				Temperature series			
Laser power	$g^{(2)}(0)_{\text{fit}}$	$g^{(2)}(0)_{\text{raw}}$	$\mathcal{P}$	Temperature	$g^{(2)}(0)_{\text{fit}}$	$g^{(2)}(0)_{\text{raw}}$	$\mathcal{P}$
$0.3 \times P_{\text{Sat}}$	0 ($\sigma = 0.075$)	0.0188	$\left(98.1^{+1.9}_{-5.6}\right)$ %	30 K	0 ($\sigma = 0.054$)	0.0808	91.9 ± 5.4%
$0.7 \times P_{\text{Sat}}$	0 ($\sigma = 0.056$)	0.0519	$\left(94.8^{+5.2}_{-0.4}\right)$ %	50 K	0.017 ($\sigma = 0.096$)	0.0486	$\left(98.3^{+1.7}_{-7.9}\right)$ %
P_{Sat}	0 ($\sigma = 0.038$)	0.0272	$\left(97.3^{+2.7}_{-1.1}\%\right)$	80 K	0.25 ($\sigma = 0.19$)	0.299	75 ± 19%

References

(1) Gschrey, M.; Thoma, A.; Schnauber, P.; Seifried, M.; Schmidt, R.; Wohlfeil, B.; Krüger, L.; Schulze, J. H.; Heindel, T.; Burger, S.; Schmidt, F.; Strittmatter, A.; Rodt, S.; Reitzenstein, S. Highly indistinguishable photons from deterministic quantum-dot microlenses utilizing three-dimensional in situ electron-beam lithography. *Nat. Commun.* **2015**, *6*, 7662.

(2) Yang, J.; Nawrath, C.; Keil, R.; Joos, R.; Zhang, X.; Höfer, B.; Chen, Y.; Zopf, M.; Jetter, M.; Portalupi, S. L.; Ding, F.; Michler, P.; Schmidt, O. G. Quantum dot-based broadband optical antenna for efficient extraction of single photons in the telecom O-band. *Opt. Express* **2020**, *28*, 19457.

(3) Kumano, H.; Harada, T.; Suemune, I.; Nakajima, H.; Kuroda, T.; Mano, T.; Sakoda, K.; Odashima, S.; Sasakura, H. Stable and efficient collection of single photons emitted from

a semiconductor quantum dot into a single-mode optical fiber. *Appl. Phys. Express* **2016**, *9*, 032801.

(4) Gür, U. M.; Arslanagić, S.; Mattes, M.; Gregersen, N. Open-geometry modal method based on transverse electric and transverse magnetic mode expansion for orthogonal curvilinear coordinates. *Phys. Rev. E* **2021**, *103*, 033301.

(5) Lavrinenko, A. V.; Lgsgaard, J.; Gregersen, N. *Numerical Methods in Photonics*; CRC PR INC, 2014.

(6) Li, L. Formulation and comparison of two recursive matrix algorithms for modeling layered diffraction gratings. *J. Opt. Soc. Amer. A* **1996**, *13*, 1024.

(7) Balanis, C. A. *Antenna Theory: Analysis and Design*; Wiley John + Sons, 2016.

(8) Abbarchi, M.; Mastrandrea, C.; Kuroda, T.; Mano, T.; Vinattieri, A.; Sakoda, K.; Gurioli, M. Poissonian statistics of excitonic complexes in quantum dots. *J. Appl. Phys.* **2009**, *106*, 053504.

(9) Baier, M. H.; Malko, A.; Pelucchi, E.; Oberli, D. Y.; Kapon, E. Quantum-dot exciton dynamics probed by photon-correlation spectroscopy. *Phys. Rev. B* **2006**, *73*, 205321.

(10) Léger, Y.; Besombes, L.; Maingault, L.; Mariette, H. Valence-band mixing in neutral, charged, and Mn-doped self-assembled quantum dots. *Phys. Rev. B* **2007**, *76*, 045331.

(11) Belhadj, T.; Amand, T.; Kunold, A.; Simon, C.-M.; Kuroda, T.; Abbarchi, M.; Mano, T.; Sakoda, K.; Kunz, S.; Marie, X.; Urbaszek, B. Impact of heavy hole-light hole coupling on optical selection rules in GaAs quantum dots. *Appl. Phys. Lett.* **2010**, *97*, 051111.

(12) Tonin, C.; Hostein, R.; Voliotis, V.; Grousson, R.; Lemaitre, A.; Martinez, A. Polarization properties of excitonic qubits in single self-assembled quantum dots. *Phys. Rev. B* **2012**, *85*, 155303.

(13) Holewa, P.; Gawełczyk, M.; Ciostek, C.; Wyborski, P.; Kadkhodazadeh, S.; Semenova, E.; Syperek, M. Optical and electronic properties of low-density InAs/InP quantum-dot-like structures designed for single-photon emitters at telecom wavelengths. *Phys. Rev. B* **2020**, *101*, 195304.

(14) https://www.lumerical.com/products/.

(15) Mrowiński, P.; Sęk, G. Modelling the enhancement of spectrally broadband extraction efficiency of emission from single InAs/InP quantum dots at telecommunication wavelengths. *Phys. B* **2019**, *562*, 141–147.

(16) Takemoto, K.; Takatsu, M.; Hirose, S.; Yokoyama, N.; Sakuma, Y.; Usuki, T.; Miyazawa, T.; Arakawa, Y. An optical horn structure for single-photon source using quantum dots at telecommunication wavelength. *J. Appl. Phys.* **2007**, *101*, 081720.

(17) Dusanowski, Ł.; Gawełczyk, M.; Misiewicz, J.; Höfling, S.; Reithmaier, J. P.; Sęk, G. Strongly temperature-dependent recombination kinetics of a negatively charged exciton in asymmetric quantum dots at 1.55 μm. *Appl. Phys. Lett.* **2018**, *113*, 043103.

(18) Syperek, M.; Dusanowski, Ł.; Andrzejewski, J.; Rudno-Rudziński, W.; Sęk, G.; Misiewicz, J.; Lelarge, F. Carrier relaxation dynamics in InAs/GaInAsP/InP(001) quantum dashes emitting near 1.55 μm. *Appl. Phys. Lett.* **2013**, *103*, 083104.

(19) Musiał, A.; Holewa, P.; Wyborski, P.; Syperek, M.; Kors, A.; Reithmaier, J. P.; Sęk, G.; Benyoucef, M. High-Purity Triggered Single-Photon Emission from Symmetric Single InAs/InP Quantum Dots around the Telecom C-Band Window. *Adv. Quantum Technol.* **2019**, *3*, 1900082.

(20) Dalgarno, P. A.; McFarlane, J.; Brunner, D.; Lambert, R. W.; Gerardot, B. D.; Warburton, R. J.; Karrai, K.; Badolato, A.; Petroff, P. M. Hole recapture limited single photon generation from a single n-type charge-tunable quantum dot. *Appl. Phys. Lett.* **2008**, *92*, 193103.

(21) Benyoucef, M.; Yacob, M.; Reithmaier, J. P.; Kettler, J.; Michler, P. Telecom-wavelength (1.5 μm) single-photon emission from InP-based quantum dots. *Appl. Phys. Lett.* **2013**, *103*, 162101.

(22) Dusanowski, Ł.; Syperek, M.; Misiewicz, J.; Somers, A.; Höfling, S.; Kamp, M.; Reithmaier, J. P.; Sęk, G. Single-photon emission of InAs/InP quantum dashes at 1.55 μm and temperatures up to 80 K. *Appl. Phys. Lett.* **2016**, *108*, 163108.

(23) Arakawa, Y.; Holmes, M. J. Progress in quantum-dot single photon sources for quantum information technologies: A broad spectrum overview. *Appl. Phys. Rev.* **2020**, *7*, 021309.

(24) Carmesin, C. et al. Structural and optical properties of InAs/(In)GaAs/GaAs quantum dots with single-photon emission in the telecom C-band up to 77 K. *Phys. Rev. B* **2018**, *98*, 125407.

(25) Lambkin, J. D.; Dunstan, D. J.; Homewood, K. P.; Howard, L. K.; Emeny, M. T. Thermal quenching of the photoluminescence of InGaAs/GaAs and InGaAs/AlGaAs strained-layer quantum wells. *Appl. Phys. Lett.* **1990**, *57*, 1986–1988.

(26) Gammon, D.; Snow, E. S.; Shanabrook, B. V.; Katzer, D. S.; Park, D. Homogeneous Linewidths in the Optical Spectrum of a Single Gallium Arsenide Quantum Dot. *Science* **1996**, *273*, 87–90.

(27) Moody, G.; Siemens, M. E.; Bristow, A. D.; Dai, X.; Karaiskaj, D.; Bracker, A. S.; Gammon, D.; Cundiff, S. T. Exciton-exciton and exciton-phonon interactions in an interfacial GaAs quantum dot ensemble. *Phys. Rev. B* **2011**, *83*, 115324.

(28) Ortner, G.; Yakovlev, D. R.; Bayer, M.; Rudin, S.; Reinecke, T. L.; Fafard, S.; Wasilewski, Z.; Forchel, A. Temperature dependence of the zero-phonon linewidth in InAs/GaAs quantum dots. *Phys. Rev. B* **2004**, *70*, 201301.
